# Consideration of health inequity in systematic reviews and primary studies on risk factors for hearing loss

**DOI:** 10.1002/cesm.12052

**Published:** 2024-04-03

**Authors:** Simon Briscoe, Elizabeth Shaw, Michael Nunns, Hassanat Lawal, Noreen Orr, Jo Thompson Coon, Ruth Garside, G. J. Melendez‐Torres

**Affiliations:** ^1^ Exeter PRP Evidence Review Facility, University of Exeter Medical School University of Exeter Exeter UK

**Keywords:** health equity, health inequalities, hearing loss, observational studies, systematic reviews

## Abstract

**Background:**

Health inequities are systematic, avoidable, and unfair differences in health between populations or population subgroups. There is increased recognition of the need for systematic reviews (SRs) to address health inequities, including drawing out findings relevant to low‐ and middle‐income countries (LMICs). The aim of this study was to determine the extent to which SRs on risk factors for hearing loss reported findings associated with health inequities, and the extent to which this data was captured in the primary studies included within these SRs.

**Methods:**

We identified SRs on risk factors for hearing loss from a report on this topic which included a systematic search for relevant SRs. SRs thus identified were inspected for data related to health inequity with reference to PROGRESS‐Plus. We compared how data were reported in SRs versus within primary studies included in the SRs, and the extent to which primary studies from LMICs were represented.

**Results:**

We included 17 SRs which reported findings on a variety of physiological, behavioral, demographic, and environmental risk factors for hearing loss. There were 296 unique primary studies included in the SRs, of which 251 (81.49%) were successfully retrieved. Data relating to health inequities was reported relatively infrequently in the SRs and mainly focused on gender and age. Data related to health inequities was more frequently reported in primary studies. However, several PROGRESS‐Plus criteria were only reported in a minority of primary studies. Approximately one‐third of primary studies were from LMICs.

**Conclusions:**

There is scope to improve the reporting of data relating to health inequities in primary studies on risk factors for hearing loss. However, SR authors could do more to report health inequities than is currently undertaken, including drawing out findings relevant to LMICs where data are available.

## 1 INTRODUCTION

The World Health Organisation (WHO) defines health inequalities as measurable differences in health between populations or population subgroups [1]. This can include differences relating to socioeconomic factors (e.g., income), geography (e.g., rural vs. urban populations), and demographic characteristics such as gender, age, and disability [2]. Some health inequalities are generally accepted as unavoidable; however, there are also health inequalities which are systematic, avoidable, and unfair differences in health between populations or population subgroups [3]. These are sometimes defined as health inequities to distinguish them from health inequalities more broadly [3]. Health inequalities which are also health inequities, can arise due to unequal treatment (e.g., systemic racism or gender bias) or wider social determinants of health, such as access to nutritious food, adequate housing, and education and employment opportunities [4]. In some cases, health interventions can themselves lead to health inequity, for example, where take up of interventions is lower within a population subgroup due to poor signposting or dissemination [5]. Health inequities are a global phenomenon which are prevalent across low‐ and middle‐income countries (LMICs) and high‐income countries [6]. Policy makers and health care commissioners acknowledge a responsibility to address health inequity alongside efforts to improve health care interventions and population health more broadly [4, 7, 8, 9]. Global initiatives such as the WHO Special Initiative for Action on Social Determinants of Health for Advancing Health Equity call for governments and international organizations to do more to address the causes of inequity and to find solutions [10].

Systematic reviews (SRs) are increasingly used to support evidence‐based policy making [11, 12, 13]. Correspondingly, the last 15 years has seen an increased recognition of the need for SRs to address health inequity in the analysis of findings, including the development of methodological guidance on how to achieve this: a recent mapping review identified 20 guidance documents published between 2009 and 2016 which develop and test a framework for addressing health inequity in SRs [14]. Several of these guidance documents refer to the PROGRESS‐Plus framework, which sets out factors which stratify health opportunities and outcomes (see Table 1) [15]. Perhaps most prominently, PROGRESS‐Plus has been used to inform the development of the PRISMA‐Equity extension, which provides guidance on reporting health inequities in SRs [16]. This includes guidance that authors should consider the degree to which the findings are applicable to disadvantaged populations, including specifically LMIC populations [17]. A major new initiative by the recently established Campbell and Cochrane Equity Methods Group seeks to make this a priority for SRs [9]. However, SRs can only include data on health inequity where these data are reported in the included primary studies. To this end, health inequity data reporting is encouraged in the CONSORT equity extension for randomized controlled trials (RCTs) [18], and in ongoing work on STROBE equity reporting guidance for observational studies [19].

**Table 1:** PROGRESS‐Plus criteria.

Characteristics	Description
PROGRESS	
Place of residence	Rural, urban, inner city
Race/ethnicity/culture	Racial, ethnic, and cultural background
Occupation	Bule collar, sedentary, noise exposed, etc.
Gender	Male, female, transgender, etc.
Religion	Religious background
Education	Years of education, level of education, etc.
Socioeconomic status	Income, type of housing, etc.
Social capital and networks	Connectedness to friends and family for support
Plus	
Personal characteristics	Age, disability, etc.
Features of relationships	Smoking parents, excluded from school, etc.
Time‐dependent relationships	Respite care, leaving the hospital, periods of time when someone is at a disadvantage

Despite recommendations that investigators include analysis of health inequity in primary and secondary research, there is still scope for improvement. Tugwell et al. [20] found that PROGRESS‐Plus relevant data was rarely reported in SRs on musculoskeletal topics despite the availability of relevant albeit limited data in the included primary studies. Basirat et al. [21] found that PROGRESS‐Plus criteria was rarely reported in either SRs on urolithiasis or in the included primary studies. Evans et al. found that SRs on eye and vision‐related topics rarely reported data on PROGRESS‐Plus criteria, however, two reviews in their sample included data extraction categories for PROGRESS‐Plus criteria for which no primary studies were found to report relevant data [22]. Thus, SR authors are sometimes hindered from considering potential health inequity by the limited detail reported in primary studies [22]. No SRs included studies conducted in LMICs in the sample considered by Tugwell et al. [20], whereas in the sample of SRs considered by Evans et al., 80% included studies conducted in LMICs, and 30% included more than half of studies conducted in LMICs [22]. Tugwell et al. propose that a lack of relevant primary studies in LMICs should be noted in the limitations of an SR as the findings may not be generalizable for LMICs [20]. Notably, Basirat et al., Evans et al., and Tugwell et al. focus on the reporting of health inequity in Cochrane reviews, none of which included observational study designs [20, 21, 22].

One area where health outcomes have been shown to closely align with wider social determinants is that of hearing loss. Hearing loss is associated with occupation [23], gender [24], educational attainment [25, 26] (including, health literacy [27]), income [28], and intellectual disabilities [29, 30]. Furthermore, the uptake of hearing loss interventions and screening programs is lower among some population subgroups, including ethnic minorities [31]. Globally, the burden of moderate to severe hearing loss is higher in LMICs than high‐income countries [32]. The reasons why hearing loss and intervention uptake are stratified by population characteristics such as these are likely to be complex and overlapping; for example, lower educational attainment may lead to working in noisy environments, or lower income may lead to limited access to health insurance [27]. Once hearing loss has occurred, this can lead to limited employment opportunities and social participation more broadly [27]. Given that hearing loss and health inequities are closely related, there is a need to understand which population subgroups are most at risk to more effectively target screening programs and interventions [33]. SRs can contribute to this by identifying, appraising, and synthesizing relevant evidence to reach more robust conclusions than otherwise possible [9, 17]. The aim of this study was to determine the extent to which SRs of risk factors for hearing loss report findings associated with health inequity. We sought to achieve this aim by addressing the following three objectives:

1. Determine whether SRs on risk factors for hearing loss and the included primary studies report baseline data relating to health inequities.
2. Determine whether SRs on risk factors for hearing loss and the included primary studies report findings relating to health inequities.
3. Determine how many primary studies included in SRs were carried out in LMICs, and whether data from LMICs were considered separately from high‐income countries in the findings of SRs.

The SRs in the study sample all included observational studies, which to the best of our knowledge, is a type of study design which has not previously been investigated for PROGRESS‐Plus criteria in the context of SR reporting.

## 2 METHODS

### 2.1 Study design and setting

This study is a secondary analysis of SR data collected for a report commissioned and funded by the UK National Institute for Health Research Policy Research Programme (NIHR200695—Evidence review facility to support national policy development and evaluation) [33]. No ethics approval was required.

### 2.2 Eligibility criteria

We included SRs which assessed all types of risk for hearing loss including environmental, behavioral, demographic, and physiological risk factors. We excluded SRs of genetically determined risk factors which were analyzed at the molecular level rather than in terms of the manifestation of disease, and adverse events arising from medical interventions. We used the UK Centre for Reviews and Dissemination's Database of Abstracts of Reviews of Effects (DARE) criteria to assess whether identified studies met sufficient criteria to be classified as SRs [34]. The publication date of included SRs was restricted from 2021 to date of search (October 2022) which provided a sufficient sample of studies to identify consistent patterns in the extent of health equity reporting in SRs compared to the included primary studies. Primary studies in the SRs were included in the analysis of equity data if they were English language journal articles from any publication date. However, we did not limit the analysis of the number of identified studies conducted in LMICs by language as this could be ascertained from bibliographic database meta‐data and freely available English language abstracts.

### 2.3 Data collection

We identified relevant SRs by inspecting Briscoe et al. which included a systematic search for SRs on risk factors for hearing loss [35]. The search was carried out in October 2022 in ASSIA (via ProQuest), Embase, HMIC and MEDLINE (all via Ovid), and Epistemonikos (https://www.epistemonikos.org/). For the purpose of this secondary analysis we also ran the search in the Cochrane Database of Systematic Reviews to check no Cochrane reviews had been missed. The MEDLINE search is reproduced in Supporting Information material (File S1). In addition, we undertook full‐text retrieval of primary studies included in relevant SRs. Data extraction forms were used to collect data from relevant SRs and primary studies. For SRs this included: risk factors considered, type of hearing loss, the number of included studies, and data relating to health inequities at baseline and results. For primary studies we extracted data related to health inequities at baseline and results, and the country setting. PROGRESS‐Plus criteria was used to categorize findings relating to health inequities [15]. We sought equity data in the included studies by inspecting the methods and results sections, and we considered that a study reported equity data at baseline if it included a breakdown of relevant characteristics, and in the results if equity relevant data were considered in the presentation of findings.

### 2.4 Data analysis and presentation

SRs were categorized by risk factor, namely behavioral, demographic, environmental, or physiological risk factors [36]. PROGRESS‐Plus relevant data at baseline and in the results of SRs and primary studies was charted numerically and presented within tables [15]. Observational studies were analyzed separately to experimental studies. We separated the analysis for these study designs because equity reporting in experimental studies, specifically RCTs, has been previously documented, whereas there is no research on equity reporting in observational studies [20, 21, 22]. Additionally, there is guidance on reporting equity for RCTs in the form of the CONSORT equity extension [18], whereas currently, there is no guidance for observational studies although this is under development [19]. Consequently, we might expect reporting to be different. Countries were classified as high‐income or LMIC with reference to the World Bank grading system and studies carried out in LMICs were charted numerically [37]. The findings of SRs were inspected to identify whether, and the extent to which, data from studies conducted in LMICs were presented separately to studies conducted in high‐income countries.

### 2.5 Patient and public involvement

We met via a Zoom videocall with a patient and public involvement (PPI) group consisting of 12 people on July 5, 2023 for 45 min to discuss their experiences and perspectives of risk factors for hearing loss, and how these relate to the findings of our analysis. This helped to interpret the findings in a wider context. The PPI group consists of a diverse group of people from across England.

## 3 RESULTS

### 3.1 Characteristics of included studies

Of the 64 SRs included in Briscoe et al. [35], 17 met the inclusion criteria for this secondary analysis (see Table 2) [38, 39, 40, 41, 42, 43, 44, 45, 46, 47, 48, 49, 50, 51, 52]. Of these, seven were published in 2021, and 10 were published in 2022. There were no relevant Cochrane reviews identified. The total number of primary studies in the included SRs was 308, of which six were included in two SRs. Thus, there were 302 unique primary studies. Of these, 251 (81.49%) were successfully retrieved at full‐text for inspection of PROGRESS‐Plus criteria [15]. The remaining 51 were either non‐English language studies, conference abstracts, or were not successfully retrieved. Thus, there were 17 SRs and 251 primary studies included in the analysis.

**Table 2:** Characteristics of included systematic reviews.

Study	Risk factor	Type of hearing loss	Population	Included studies, *n* (FT retrieved, % of total)
Physiological risk factors				
Beukes et al. [38]	COVID‐19	Tinnitus, conductive hearing loss, SSNHL, hearing loss (unspecified)	All age groups experiencing tinnitus	33 (32, 97%)
Elizinga et al. [39]	Otitis media	SNHL	All age groups with otitis media	9 (8, 88.89%)
Frosolini et al. [40]	Inflammatory biomarkers	SSNHL	All age groups with SSNHL	13 (12, 92.31%)
Jeong et al. [41]	Psoriasis	Hearing loss (unspecified), SNHL	Individuals with psoriasis	13 (9, 69.23%)
Kapoor et al. [42]	Sickle cell disease	Hearing loss (unspecified)	Adults with sickle cell disease	12 (7, 58.33%)
Kasemsuk et al. [43]	Obstructive sleep apnea	Hearing loss (unspecified)	Individuals with obstructive sleep apnea	20 (19, 95%)
Lien et al. [44]	Vitiligo	High‐frequency SNHL	Individuals with vitiligo	9 (7, 77.77%)
Meng et al. [45]	COVID‐19	SSNHL	Individuals with COVID‐19	26 (25, 96.15%)
Mirmosayyeb et al. [46]	Multiple sclerosis	SNHL	Individuals with MS and hearing loss	8 (6, 75%)
Paraschou et al. [47]	Systemic lupus erythematosus	SNHL, conductive hearing loss, mixed hearing loss	Individuals with systemic lupus erythematosus	9 (6, 66.67%)
Behavioral risk factors				
Taziki Balajelini et al. [48]	Mobile phone use	Hearing loss (unspecified)	Mobile phone users	4 (3, 75%)
Demographic risk factors				
Dawes et al. [49]	Early life influences including birth weight and adult height	Adult‐onset hearing loss (unspecified)	Adults	8 (6, 75%)
Raeisi et al. [50]	Multiple factors	Hearing loss (unspecified)	Newborns	18 (18, 100%)
Environmental risk factors				
Basu et al. [51]	Occupational noise	Hearing loss (unspecified)	Workers	21 (21, 100%)
Dineva et al. [52]	Iodine exposure	Hearing loss, hearing acuity, binaural processing skills, binaural memory, auditory memory, infant hearing	Pregnant women and children	13 (7, 53.85%)
Meghji and Phillips [53]	Noise exposure	Noise‐induced hearing loss (sensorineural hearing loss)	Adults and children with exposure to noise	84 (63, 75%)
Yin et al. [54]	Lead exposure	Hearing loss PTA > 25 dB	All ages	8 (8, 100%)

**Figure 1:**
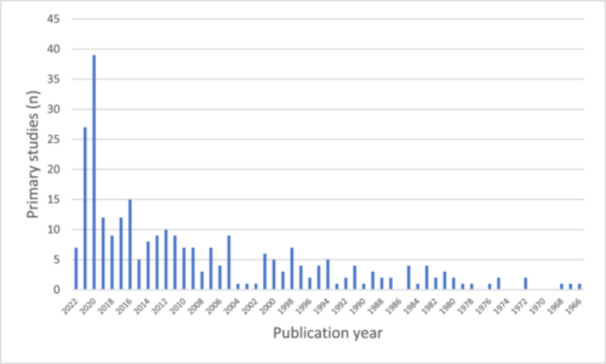
Primary studies included in systematic reviews per year of publication.

Of the 17 included SRs, 10 considered physiological risk factors for hearing loss [38, 39, 40, 41, 42, 43, 44, 45, 46, 47], four considered environmental risk factors [52, 53, 54], two considered demographic risk factors [49, 50], and one considered behavioral risk factors [48]. Of the 251 primary studies, 248 were observational studies, and three were RCTs. Dineva et al. included two RCTs [52], and Jeong et al. included one RCT [41]. The observational studies consisted of 104 cohort studies, 59 case control studies, 55 cross‐sectional studies, 20 case studies, and 10 case series. The median number of primary studies included in the SRs was 13 (range = 4–84). The median date of publication of included primary studies was 2014 (range = 1966–2022) and was skewed towards studies published since 2000 (see Figure 1).

### 3.2 PROGRESS‐Plus criteria at baseline and results in SRs

**Figure 2:**
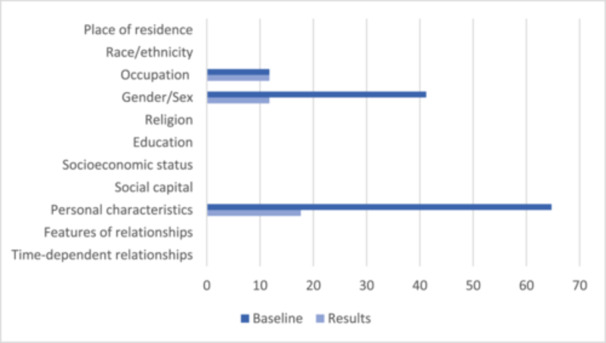
Percentage of systematic reviews reporting PROGRESS‐Plus criteria at baseline and in results.

The most frequently reported PROGRESS‐Plus relevant data in the included SRs at baseline and in the analysis of results were personal characteristics associated with discrimination, the majority of which related to the age of participants (see Figure 2). A total of 11 reviews (64.71%) reported personal characteristics associated with discrimination at baseline (all relating to age) [38, 39, 40, 41, 43, 44, 47, 49, 51, 52, 53], and three reviews (17.65%) reported data relating to personal characteristics associated with discrimination in the analysis of results (including one review which reported disability and two which reported age) (see Table 3) [38, 50, 53]. Less than half of SRs reported the gender of participants at baseline (*n* = 7, 41.18%) [38, 40, 41, 43, 47, 49, 51], and two reported data relating to the gender of participants in the analysis of results (11.76%) (see Table 3) [38, 53]. Occupation was reported at baseline and in the analysis of results in three reviews (17.65%) [38, 51, 53]. No data was reported about other PROGRESS‐Plus criteria.

**Table 3:** Frequency of PROGRESS‐Plus criteria reported in systematic reviews and included observational studies at baseline and results.

PROGRESS‐Plus	Baseline data		Results data	
	SRs, *n* (%)	Observational studies, *n* (%)	SRs, *n* (%)	Observational studies, *n* (%)
Place of residence	0 (0)	9 (3.63)	0 (0)	7 (2.82)
Race/ethnicity	0 (0)	23 (9.27)	0 (0)	10 (4.03)
Occupation	2 (11.76)	81 (32.66)	2 (11.76)	69 (27.82)
Gender/sex	7 (41.18)	209 (84.27)	2 (11.76)	108 (43.55)
Religion	0 (0)	0 (0)	0 (0)	0 (0)
Education	0 (0)	16 (6.45)	0 (0)	13 (5.24)
Socioeconomic status	0 (0)	10 (4.03)	0 (0)	8 (3.22)
Social capital	0 (0)	1 (0.4)	0 (0)	0 (0)
Personal characteristics^a^	11 (64.71)^d^	212 (85.48)	3 (17.65)	101 (40.73)
Features of relationships^b^	0 (0)	8 (3.22)	1 (5.88)	3 (1.21)
Time‐dependent relationships^c^	0 (0)	0 (0)	0 (0)	0 (0)

^a^ Personal characteristics associated with discrimination (e.g., age, disability).

^b^ Features of relationships (e.g., smoking parents, excluded from school).

^c^ Time dependent relationships (e.g., leaving the hospital, respite care).

^d^ All personal characteristics data reported at baseline in systematic reviews relate to age.

Four SRs (23.53%) reported PROGRESS‐Plus relevant data in the analysis of the results [38, 50, 51, 53]. Specifically, Basu et al. reported findings on occupation‐induced hearing loss, including detail on hearing loss experienced amongst a variety of workers [51]. Beukes et al. reported that tinnitus was found to be more bothersome during the COVID‐19 pandemic for females, adults under the age of 50, and was exacerbated by self‐isolation and loneliness [38]. Meghji et al. reported findings on occupational noise exposure, and in particular, found that males and older persons were at higher risk of hearing loss [53]. Raeisi et al. found that infants with disabilities, including developmental delay and craniofacial anomalies, were at higher risk of hearing loss [50]. A breakdown of which PROGRESS‐Plus criteria were reported within each included SR and the corresponding included primary studies across both observational and experimental study designs at baseline and in the results is reported in Additional File 2.

### 3.3 PROGRESS criteria at baseline and results in primary studies

#### 3.3.1 Observational studies

**Figure 3:**
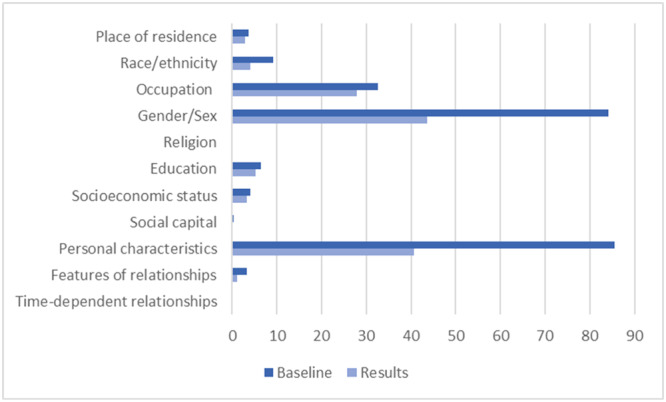
Percentage of observational studies reporting PROGRESS‐Plus criteria at baseline and in results.

In the 248 observational studies included in the analysis, data relating to gender and personal characteristics (predominantly age) were reported at baseline in around 85% of studies (see Table 3). Data relating to these criteria were reported in the analysis of the results in approximately half of these studies (43.55% and 40.73% for gender and personal characteristics, respectively). The occupation was the next most frequently reported data, including at baseline in 81 studies (32.66%) and in the results of 69 studies (27.82%) (see Table 3). All 17 SRs included studies which reported gender and personal characteristics at baseline, and 15 SRs included studies which reported gender and personal characteristics in the analysis of results, although in some reviews the latter was in a small proportion of included studies [38, 41, 42, 43, 44, 45, 46, 47, 48, 49, 50, 51, 52, 53, 54]. Studies reporting data on occupation were included in a total of eight SRs [38, 42, 45, 48, 49, 51, 53, 54]. Place of residence, race/ethnicity, education, socioeconomic status, social capital and features of relationships were also reported across the included studies, albeit in fewer than 10% of studies at both baseline and in results for all criteria. Across all the criteria, baseline data was reported more frequently than results data (see Figure 3).

The reporting of data relating to gender and characteristics associated with discrimination (mainly, age) were relatively uniform across observational studies, for example, the studies typically reported the same gender categories, specifically, male and female. A small number of studies reported cisgender and transgender categories. The reporting of data relating to other PROGRESS‐Plus criteria was more varied. The categories White, Black, and Asian were commonly used for race/ethnicity, but other categories were also sometimes used alongside these depending on the geographical region, for example, Mexican or Hispanic. Categories for place of residence were sometimes divided between rural and urban, or sometimes included more gradation between different types of urban settings (e.g., inner city). Educational attainment and socioeconomic status categories were similarly varied, often according to geographical region.

#### 3.3.2 Experimental studies

The pattern of reporting of PROGRESS‐Plus criteria was similar in the three experimental studies included in the analysis as the observational studies [55, 56, 57]. All three studies reported personal characteristics associated with health inequity at baseline (specifically, age), and two reported gender at baseline [55, 57]. Across the three studies, detail was also reported about education at baseline, age at results, and features of relationships associated with health inequity.

### 3.4 Primary studies set in LMICs

A total of 118 primary studies (38.31%) across the 17 included SRs were carried out in LMICs. Only one SR did not include any primary studies set in LMICs [49] and in one review all of the primary studies were set in LMICs [51]. Other than in Basu et al. which included solely primary studies set in LMICs [51], four SRs included a majority of primary studies set in LMICs [39, 44, 48, 50]. Kapoor et al. presented the most extensive analysis of findings by geographical region, including analyses of data from Africa, Europe, the Middle East, North America, and South America [42]. Although this breakdown was not explicitly grouped according to LMICs, it did allow for consideration of findings in global regions where LMICs are more prevalent. Typically, SRs presented data on the characteristics of included studies which included the country setting for each study, but did not draw out findings which are relevant to LMICs in the syntheses. Other than Kapoor et al., the exceptions were SRs, where data for one or more parts of the synthesis drew exclusively on studies conducted in LMICs. However, unlike Kapoor et al., these examples appeared to solely arise due to only identifying relevant data from studies conducted in LMICs rather than by design [42].

## 4 DISCUSSION

The findings of this study show that few criteria associated with health inequity were considered in our sample of SRs on risk factors for hearing loss. The findings also show that primary studies included in the SRs reported more data associated with health inequity than is reported in the SRs, both in terms of the type and frequency of PROG RESS‐Plus relevant data that was reported. However, although overall the primary studies reported more types of PROGRESS‐Plus relevant data (including place of residence, race/ethnicity, and education) this was in a minority of studies. Thus, the lack of reporting of these criteria in SRs may reflect that there was limited scope for data analysis within any one SR for these criteria. Although the sample size of SRs was small, it was sufficient to identify patterns in the reporting of health equity data in SRs and primary studies. Studies conducted in LMICs were included in the majority of SRs, but data from LMIC studies were not typically considered separately to data from studies in high‐income countries.

Our findings are similar to those of studies which investigate the reporting of health inequity in Cochrane reviews of clinical trials [20, 21, 22]. Basirat et al. found that the only PROGRESS data reported in a sample of 12 Cochrane reviews published 2012–2022 on interventions for kidney stones related to gender (*n* = 2, 16.7%) and place of residence (*n* = 1, 8.3%) [21]. In contrast, in the 130 included primary studies, data related to these categories was reported more frequently (92.9% and 25% of studies, respectively). It is not clear whether this was at baseline or in the results. Tugwell et al. found that gender and place of residence were the only PROGRESS data reported at baseline in a sample of 14 Cochrane reviews on musculoskeletal conditions published 2003–2007. Gender was reported in five reviews (35.7%) and place of residence in two reviews (14.3%). In comparison, baseline data on gender was reported in 89.9% of primary studies (*n* = 131) and on place of residence in 17.7% of studies (*n* = 20). Furthermore, primary studies also reported race/ethnicity (*n* = 26, 17.7%), occupation (*n* = 24, 16.3%), education (n = 36, 24.5%), socioeconomic status (n = 15, 10.2%) and social capital (n = 23, 15.6%) at baseline [20]. No SRs in the sample reported PROGRESS criteria in the results versus eleven primary studies [20].

Evans et al. also found that gender was more frequently reported at baseline in primary studies (*n* = 45, 73%) than SRs (*n* = 12, 52%) in a sample of 24 Cochrane reviews on eye and vision topics published 2013‐2019 [22]. Atypically, the percentage of reviews which considered health inequity overall was higher than in the included studies, which owes to two SRs which set out to capture a range of PROGRESS‐Plus criteria which were not reported in the included primary studies [22]. No SRs considered health inequity in the analysis of findings [22].

The proportion of primary studies set in LMICs in the present study was similar to that reported in Evans et al. (38% and 37%, respectively) [22]. (As noted above, Tugwell et al. reported no primary studies were set in LMICs [20]; Basirat et al. reported “very few” studies were set in LMICs [21]). The proportion of studies conducted in LMICs in the present study can be considered low given that the age‐standardized burden of moderate to severe hearing loss globally is higher in LMICs than high‐income countries [32]. Consideration of data from studies conducted in LMICs separately to data from studies conducted in high‐income countries was limited. As an illustration of the value of drawing out data specific to LMICs in the findings on risk factors for hearing loss, the WHO global region with the greatest predicted percentage increase in moderate to complete hearing loss by 2050 is the African region (154%) [32]. This is also the region with the lowest availability of audiologists who have the skills to diagnose and address hearing loss, with fewer than 1 audiologist per 1 million people in 78% of African countries, compared to 10 audiologists per 1 million people in high‐income countries [58]. Thus, there is an intensified need for identification of risk factors for hearing loss in the African region which can lead to preventative measures.

### 4.1 Patient and public involvement

It was noted in the PPI group discussion that the incidence of hearing loss has increased since the COVID‐19 pandemic, and that this was unlikely to be reflected in the findings of most SRs which include primary studies pre‐dating the pandemic. Thus, it was noted there is now an increased need to address health inequity related to risk factors for hearing loss. It was also noted that hearing loss can be a gradual process, and that people experiencing hearing loss are sometimes either reluctant to seek help or unaware of the available help, during which time hearing loss may become more severe. In some cases, perceived stigma associated with hearing loss can delay uptake of services. Immigrant populations who experience hearing loss may be unaware that they can obtain support via the NHS if this is not possible in their country of birth. Family members, carers, and friends were identified as important in signposting people at risk of hearing loss to health and social care services, but it was also noted that in some communities there is a widespread lack of awareness of available support. These examples show how subgroup populations who are at risk for hearing loss may experience delays to obtaining appropriate support.

### 4.2 Implications for practice

Observational studies are particularly suited to identifying long‐term health trends, within which health inequities are important to consider [59, 60]. The findings of this study show that both observational studies and SRs on risk factors for hearing loss need to be more diligent in the reporting of data related to health inequity. Although the number of identified studies conducted in LMICs may be fewer in number than the number conducted high‐income countries, there is still scope for SR authors to do more to draw out findings which are specific to LMICs where data are available, and to highlight country‐specific gaps in the evidence for future research to address. Initiatives such as the Campbell and Cochrane Equity Methods Group can contribute to this through raising awareness [9]. The few SRs in the sample which did report data associated with health inequity in the results were able to draw out findings which may be relevant to health care providers and policy makers, concerning gender, occupation, age, and social capital [38, 50, 51, 53]. The discussion with the PPI group further shows that risk factors for hearing loss are potentially experienced differently between population subgroups. The relatively frequent reporting of data on occupation in primary studies in the present study, compared with other types of PROGRESS‐Plus relevant data in the primary studies (aside from gender or age) or in similar studies to date [20, 21, 22], may reflect that occupation is particularly relevant to hearing loss in the form of noise exposure and should be considered in this context [23]. The PRISMA‐Equity extension recommends identifying which PROGRESS‐Plus criteria are relevant to the phenomenon of interest; occupation may be a prominent example here [16]. Nonetheless, evidence and discussion with the PPI group suggests that inequities associated with risk factors for hearing loss are more wide ranging than this [23, 24, 25, 26, 27, 28, 29, 31, 61]. The insight provided by our PPI group suggests that if researchers plan to focus on specific equity criteria when carrying out an SR, a preliminary discussion with a PPI group with relevant experience could be invaluable for identifying relevant criteria.

The PRISMA‐Equity framework notes that data related to PROGRESS‐Plus criteria are likely to be described differently across studies [16]. This was corroborated in the primary studies which reported data on race/ethnicity, place of residence, socioeconomic status, and educational attainment. The PRISMA‐Equity framework recommends that careful consideration is given to their definition and classification for the purpose of an SR, in addition their impact on health inequity. A degree of interpretation is also required in considering what the implications are for any health inequalities identified in the analysis of findings of SRs, and how these relate to inequity.

### 4.3 Strengths and limitations

To the best of our knowledge, this is the first study to investigate the reporting of data related to health inequity in SRs of risk factors for hearing loss. Furthermore, we believe the study is the first to investigate health inequity reporting in SRs which includes observational study designs. A potential limitation is that because the reviews are published in different journals and have not all followed the same methodological approach (as per Cochrane reviews), the quality of the reviews and the expectations of the publishers is likely to be more varied. This may account for absence of reporting of health inequity data in some reviews. Although the sample size of SRs was small, it was sufficient to identify patterns in the reporting of health equity data in SRs and primary studies, and equivalent to similar studies on the reporting of health equity data in SRs [20, 21, 22]. The majority of primary studies in the sample were published post‐2000 and the median date of publication was 2014, however, there were still a proportion of studies published pre‐2000 which may have been less likely to include data on health inequities. Thus, the proportion of studies reporting health inequities is likely have been higher if only primary studies in the recent past were included in the analysis. We did not have resources for translating non‐English language studies which is a limitation of our analysis of equity data, but this did not affect our calculation of the number of studies conducted in LMICs for which we could use bibliographic database meta‐data and freely available English language abstracts.

## 5 CONCLUSIONS

Health inequities are not routinely considered in SRs of risk factors for hearing loss. There is a need to improve the reporting of data relating to health inequity both in primary studies and SRs, to gain an understanding of how these factors are experienced differently by population subgroups.

## AUTHOR CONTRIBUTIONS

**Simon Briscoe**: Conceptualization; data curation; formal analysis; investigation; methodology; project administration; writing—original draft; writing—review and editing. **Elizabeth Shaw**: Formal analysis; investigation; writing—review and editing. **Michael Nunns**: formal analysis; investigation; writing—review and editing. **Hassanat Lawal**: Formal analysis; investigation; writing—review and editing. **Noreen Orr**: Formal analysis; investigation; writing—review and editing. **Jo Thompson Coon**: Formal analysis; investigation; writing—review and editing. **Ruth Garside**: Formal analysis; investigation; writing—review and editing. **G. J. Melendez‐Torres**: Formal analysis; Investigation; Writing—review and editing.

## CONFLICT OF INTEREST STATEMENT

The authors declare no conflict of interest.

## PEER REVIEW

The peer review history for this article is available at https://www.webofscience.com/api/gateway/wos/peer-review/10.1002/cesm.12052.

## Supporting information

Supporting information.

Supporting information.

Supporting information.

## Data Availability

The data sets used and/or analyzed during the current study are available from the corresponding author on reasonable request.
