# Supplementary material for: Consideration of health inequity in systematic reviews and primary studies on risk factors for hearing loss
Source: Cochrane Evid Synth Methods. 2024 Apr 3;2(4):e12052. doi: 10.1002/cesm.12052 (PMC11795950; doi:10.1002/cesm.12052)
Supplement: Supplementary file 1 — Supporting information. [file CESM-2-e12052-s002.docx]

**Additional File 1.** Ovid MEDLINE search

1. ((hearing or audiolog* or acoustic or otologic*) adj3 (health or impair* or inequalit* or loss)).tw.
2. deaf*.tw.
3. exp Hearing Loss/
4. Persons With Hearing Impairments/
5. "acoustic neuroma".tw.
6. Neuroma, Acoustic/
7. or/1-6
8. exp Risk/
9. risk*.tw.
10. exp Prognosis/
11. prognosis.tw.
12. predict*.tw.
13. exp Incidence/
14. incidence.tw.
15. "causal factor*".tw.
16. epidemiolo*.tw.
17. Epidemiology/
18. or/8-17
19. (equit* or inequit* or inequalit* or disparit* or equality).tw.
20. (ethnic* or race or racial* or racis*).tw.
21. ((social* or "socio-economic" or socioeconomic or economic or structural or material) adj3 (advantage* or disadvantage* or exclude* or exclusion or include* or inclusion or status or position or gradient* or hierarch* or class* or determinant*)).tw.
22. (health adj3 (gap* or gradient* or hierarch*)).tw.
23. Vulnerable populations/
24. socioeconomic factors/
25. poverty/
26. social class/
27. Healthcare Disparities/
28. Health Status Disparities/
29. Poverty areas/
30. Urban population/
31. (SES or SEP or sociodemographic* or "socio-demographic*" or income or wealth* or poverty or "educational level" or "level of education" or "educational attainment" or "well educated" or "better educated" or unemploy* or "home owner*" or tenure or affluen* or "well off" or "better off" or "worse off").tw.
32. or/19-31
33. 18 or 32
34. ((map or mapping or rapid or systematic or scoping or umbrella) adj2 (review* or synthes*)).tw.
35. ("meta analy*" or metaanaly* or metasynthe* or "meta synthe*").tw.
36. "evidence synthes*".tw.
37. "review* of reviews".tw.
38. systematic review.pt.
39. meta-analysis.pt.
40. or/34-39
41. 7 and 33 and 40
