# Supplementary material for: Consideration of health inequity in systematic reviews and primary studies on risk factors for hearing loss
Source: Cochrane Evid Synth Methods. 2024 Apr 3;2(4):e12052. doi: 10.1002/cesm.12052 (PMC11795950; doi:10.1002/cesm.12052)
Supplement: Supplementary file 2 — Supporting information. [file CESM-2-e12052-s003.docx]

**Additional File 2.** PROGRESS-Plus criteria per systematic review and included primary studies at baseline and results

| **Study** | **Baseline data** | | **Results** | |
| --- | --- | --- | --- | --- |
|  | ***Systematic review*** | ***Primary studies*** | ***Systematic review*** | ***Primary studies*** |
| *Physiological risk factors* | | | | |
| Beukes 2021 | Occupation, gender  personal characteristics (age) | Place of residence, race/ethnicity, occupation, gender, education, socioeconomic status, personal characteristics (age) | Occupation, gender, personal characteristics (age) | Place of residence, gender, education, personal characteristics (age) |
| Elizinga 2021 | Personal characteristics (age) | Gender, personal characteristics (age) | NR | Personal characteristics (age) |
| Frosolini 2022 | Gender, personal characteristics (age) | Gender, personal characteristics (age) | NR | Gender, personal characteristics (age) |
| Jeong 2022 | Gender, personal characteristics (age) | Gender, personal characteristics (age) | NR | Gender, personal characteristics (age) |
| Kapoor 2021 | NR | Race/ethnicity, gender, gender, occupation, personal characteristics (age) | NR | Gender, personal characteristics (age) |
| Kasemsuk 2022 | Gender, personal characteristics (age) | Race/ethnicity, gender, socioeconomic status, personal characteristics (age) | NR | Race/ethnicity, gender, socioeconomic status, personal characteristics (age) |
| Lien 2022 | Personal characteristics (age) | Gender, personal characteristics (age) | NR | Gender |
| Meng 2022 | NR | Occupation, gender, social capital, personal characteristics (age) | NR | Race/ethnicity, occupation, gender, personal characteristics (age) |
| Mirmosayyeb 2022 | NR | Race/ethnicity, gender, personal characteristics (age) | NR | Gender, personal characteristics (age) |
| Paraschou 2021 | Gender, personal characteristics (age) | Place of residence, race/ethnicity, gender, socioeconomic status, personal characteristics (age) | NR | Place of residence, gender, socioeconomic status, personal characteristics (age) |
| *Behavioural risk factors* | | | | |
| Taziki Balajelini 2021 | NR | Place of residence, race/ethnicity, occupation, gender, education, socioeconomic status, personal characteristics (age) | NR | Place of residence, occupation, gender, education, socioeconomic status, personal characteristics (age) |
| *Demographic risk factors* | | | | |
| Dawes 2022 | Gender, personal characteristics (age) | Race/ethnicity, occupation, gender, socioeconomic status, personal characteristics (age) | NR | Occupation, gender, education, personal characteristics (age), features of relationships (maternal smoking) |
| Raeisi 2022 | NR | Race/ethnicity, gender, personal characteristics (age, disability), features of relationships (parental education, parental occupation) | Personal characteristics (disability) | Race/ethnicity, personal characteristics (age, disability), features of relationships (paternal education, parental occupation) |
| *Environmental risk factors* | | | | |
| Basu 2022 | Occupation, gender, personal characteristics (age) | Occupation, gender , education, socioeconomic status, personal characteristics (age) | Occupation | Occupation, gender, personal characteristics (age), |
| Dineva 2022 | Personal characteristics (age) | Place of residence, race/ethnicity, gender, education, socioeconomic status, personal characteristics (age), features of relationships (parental education, parental occupation) | NR | Place of residence, gender, socioeconomic status, personal characteristics (age) |
| Meghji 2021 | Occupation, personal characteristics (age) | Place of residence, race/ethnicity, occupation, gender, education, socioeconomic status, personal characteristics (age), features of relationships (parental education, parental income) | Occupation, gender, personal characteristics (age) | Place of residence, race/ethnicity, occupation, gender, education, socioeconomic status, personal characteristics (age) |
| Yin 2021 | NR | Race/ethnicity, occupation, gender, education, socioeconomic status, personal characteristics (age), features of relationships (parental education, parental income, parental smoking) | NR | Race/ethnicity, occupation, gender, education, socioeconomic status, personal characteristics (age), features of relationships (parental education, parental income, parental smoking) |
